# Supplementary material for: PCLIPtools: A Robust Framework for Identifying RNA-Protein Interaction Sites from PAR-CLIP experiments
Source: bioRxiv. 2025 Nov 18:2025.11.17.688892. Preprint. [Version 1] doi: 10.1101/2025.11.17.688892 (PMC12667781; doi:10.1101/2025.11.17.688892)
Supplement: Supplement 1 [file media-1.pdf]

PCLIPtools: A Robust Framework for Identifying RNA-Protein Interaction Sites from PAR-CLIP experiments.

Authors
Ahsan H. Polash and Markus Hafner
Affiliation:
RNA Molecular Biology Laboratory, National Institute of Arthritis and Musculoskeletal and Skin Diseases (NIAMS), National Institutes of Health (NIH), Bethesda, 20892 Maryland, USA

Correspondence should be addressed to **ahsan.polash@nih.gov** (**AP**) or **markus.hafner@nih.gov** (**MH**)

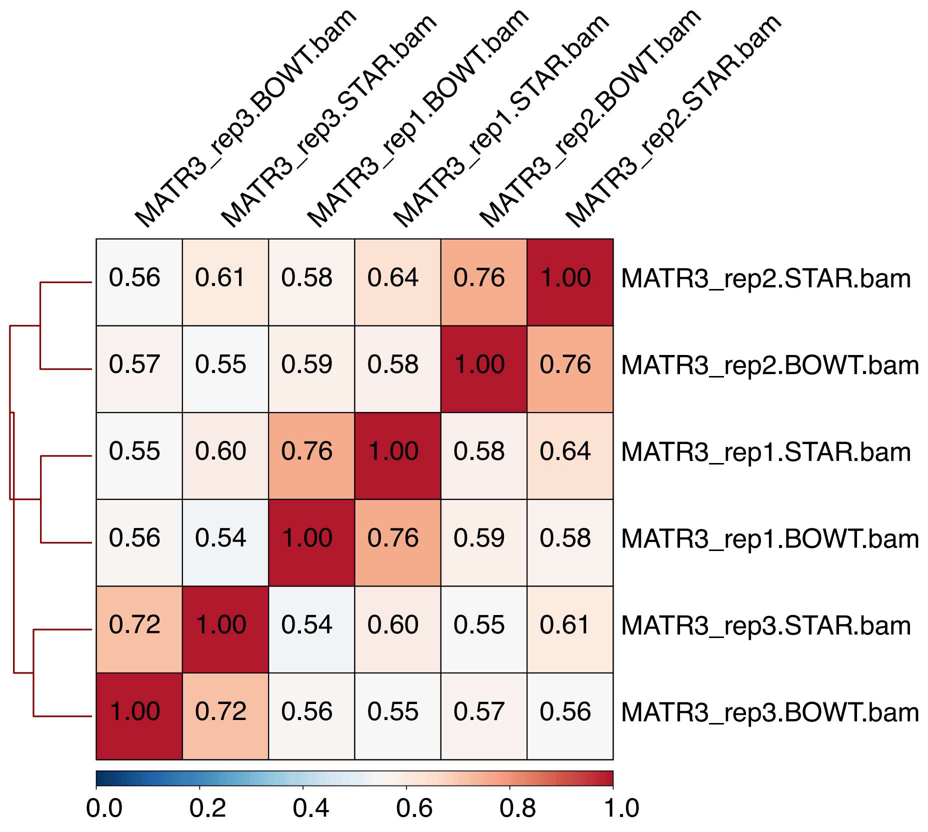

Supplementary Figure 1. Both STAR and Bowtie (BOWT) aligners resulted in comparable alignments. However, STAR aligned bam files are more uniform accross replicates.

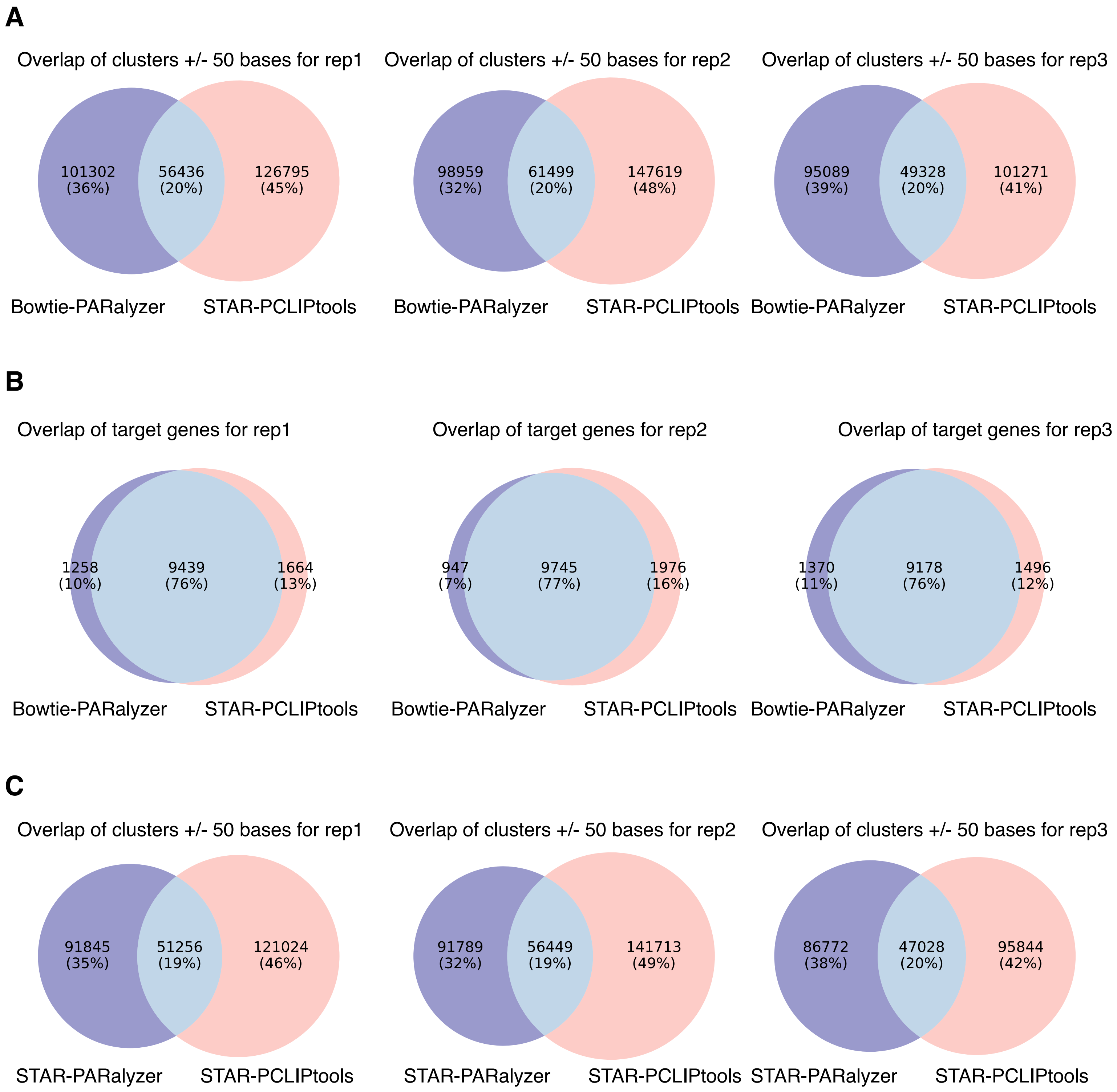

Supplementary Figure 2. Comparison of cluster coordinates (+/- 50 bases) for (**A**) Bowtie-PARalyzer and STAR-PCLIPtools, (**C**) STAR-PARalyzer and STAR-PCLIPtool showed substantial amount of clusters were unique due to the differences in the peak calling tools and the aligner tool used. (**B**) Regardless of the alignment method and the peak calling method, comparison of the PAR-CLIP target genes are more uniform than comparing the exact interaction sites.

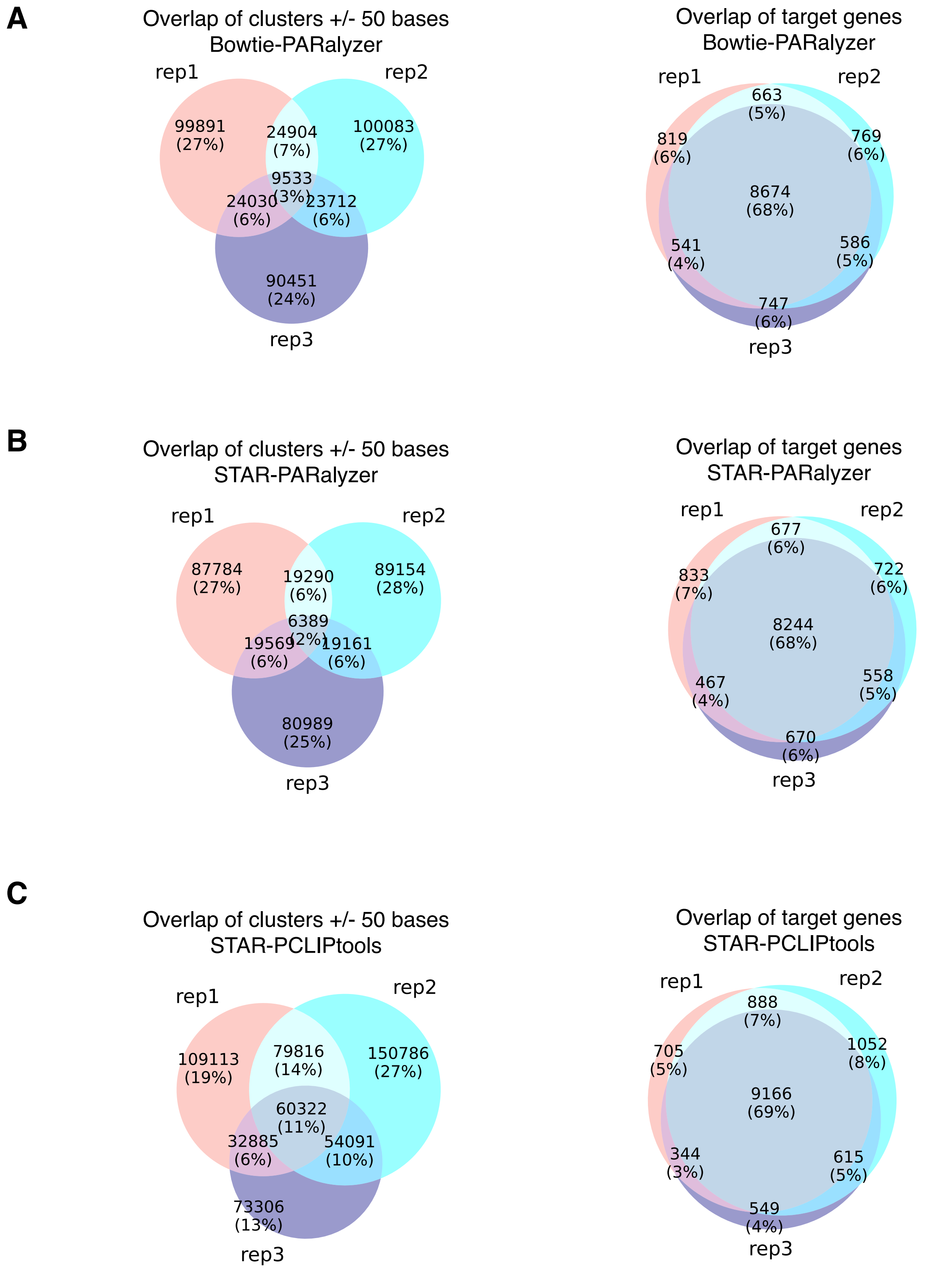

Supplementary Figure 3. Comparison of cluster coordinates (+/- 50 bases) derived from (**A**) Bowtie-PARalyzer, (**B**) STAR-PARalyzer and (**C**) STAR-PCLIPtools showed the latter resulted in higher reproducibility accross replicates compared to rest.

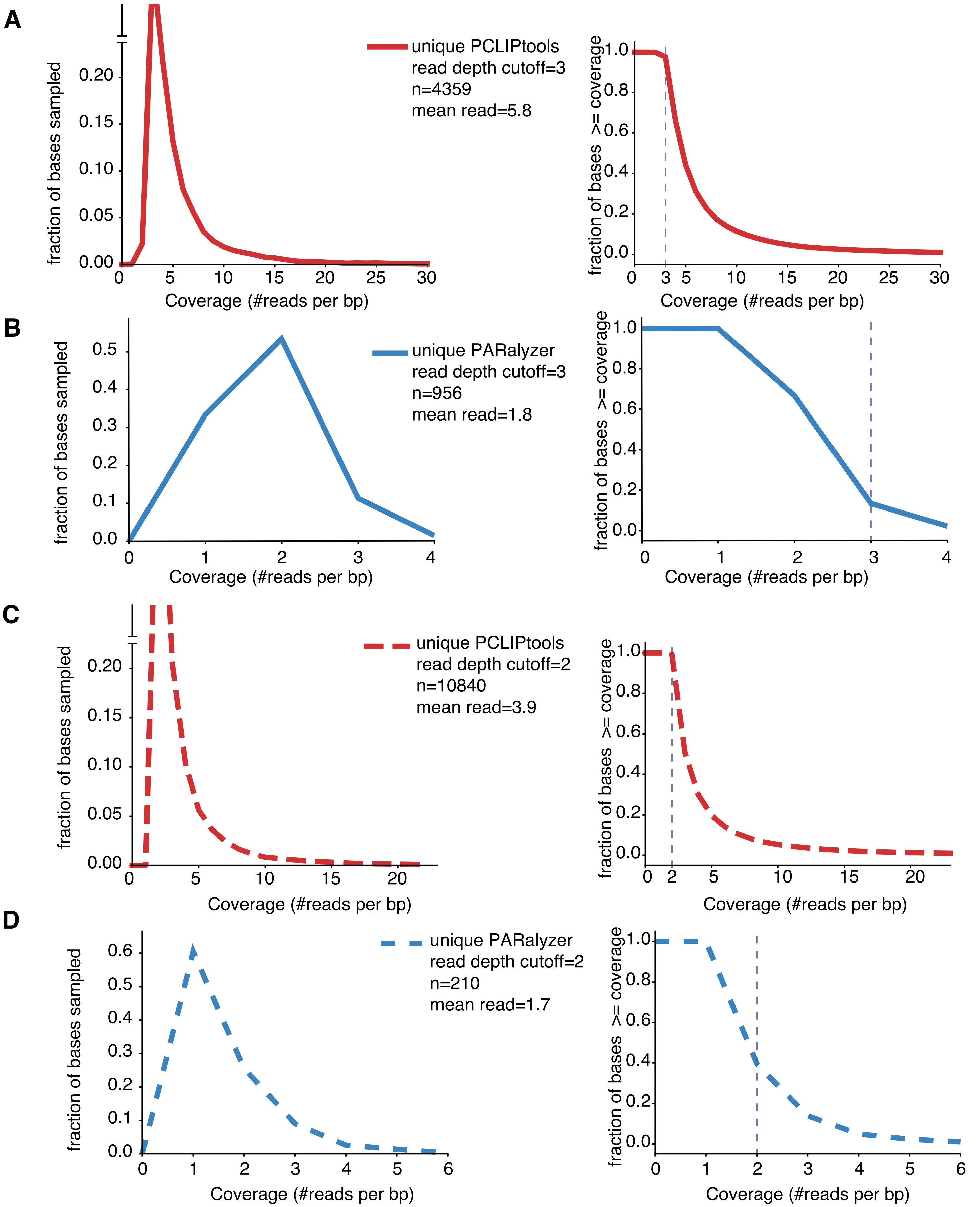

Supplementary Figure 4: PCLIPtools specific clusters for chromosome 13 are enriched with high read depth (**A** left panel) and (**C** left panel) comapred to PARalyzer spefic clusters (**B** left panel) and (**D** left panel). PCLIPtools maintains minimum read threshold 3 reads (**A** right panel) and 2 reads (**C** right panel) for clusters whereas PARalyzer often retains clusters that do not meet minimum read depth threshold (**B** right panel) and (**D** right panel).
